# Adapting Yeast as Model to Study Ricin Toxin A Uptake and Trafficking^‡^

**DOI:** 10.3390/toxins3070834

**Published:** 2011-07-05

**Authors:** Björn Becker, Manfred J. Schmitt

**Affiliations:** Molecular and Cell Biology, Department of Biosciences (FR 8.3), Saarland University, D-66041Saarbrücken, Germany; Email: bjoern_becker2@gmx.de

**Keywords:** ricin toxin A chain, ER retention signal, yeast spheroplasts, toxin endocytosis and transport, oyxgen-sensor microtiter plate, H/KDEL

## Abstract

The plant A/B toxin ricin represents a heterodimeric glycoprotein belonging to the family of ribosome inactivating proteins, RIPs. Its toxicity towards eukaryotic cells results from the depurination of 28S rRNA due to the *N*-glycosidic activity of ricin toxin A chain, RTA. Since the extention of RTA by a mammalian-specific endoplasmic reticulum (ER) retention signal (KDEL) significantly increases RTA *in vivo* toxicity against mammalian cells, we here analyzed the phenotypic effect of RTA carrying the yeast-specific ER retention motif HDEL. Interestingly, such a toxin (RTA^HDEL^) showed a similar cytotoxic effect on yeast as a corresponding RTA^KDEL^ variant on HeLa cells. Furthermore, we established a powerful yeast bioassay for RTA *in vivo* uptake and trafficking which is based on the measurement of dissolved oxygen in toxin-treated spheroplast cultures of *S. cerevisiae*. We show that yeast spheroplasts are highly sensitive against external applied RTA and further demonstrate that its toxicity is greatly enhanced by replacing the *C*-terminal KDEL motif by HDEL. Based on the RTA resistant phenotype seen in yeast knock-out mutants defective in early steps of endocytosis (∆*end3*) and/or in RTA depurination activity on 28S rRNA (∆*rpl12B*) we feel that the yeast-based bioassay described in this study is a powerful tool to dissect intracellular A/B toxin transport from the plasma membrane through the endosomal compartment to the ER.

## Abbreviations

ER: endoplasmic reticulum
RTA: ricin toxin A chain
RTB: ricin toxin B chain
PI: propidiumiodide

## 1. Introduction

The development of effective protecting drugs and antidotes against various human diseases and biological toxins is still a major goal in ongoing biomedical research. The plant *Ricinus communis* produces a heterodimeric protein toxin (ricin) which represents one of the most powerful A/B toxins of biological heritage [1]. The mature holotoxin represents a glycosylated heterodimer consisting of two polypeptide chains which are covalently linked through a single disulfide bond [2,3]. Ricin toxin A-chain (RTA; 30 kDa) acts as *N*-glycosidase by cleaving a specific adenine residue at position 4324 within the sarcin/ricin loop of 28S rRNA [4,5]. Loss of this adenine residue hesitates *in vivo* binding of elongation factor 2 (EF2) which is required to initiate protein translation on the eukaryotic 80S ribosome [6]. Thus, ricin treated cells are rapidly blocked in protein biosynthesis and subsequently committed to cell death [2]. In contrast to RTA, the 34 kDa B-chain RTB represents the cell surface binding component which mediates toxin uptake by the target cell [7]. After toxin binding to terminal galactose and/or *N*-acetylglucosamine residues on the surface of mammalian cells, the toxin is taken up by endocytosis and transported to early endosomes [7,8]. While this transport can occur both in a clathrin-dependent or clathrin-independent manner [9,10,11], only 5% of the toxin molecules finally reach the TGN network [12]. Synthaxin 5 plays an important role in this toxin transport step [13]. In contrast to other A/B toxin family members such as Cholera toxin or the *E. coli* heat labile toxin HLT [14], ricin itself does not contain an ER retention signal which could potentially mediate its retrograde transport into the ER through binding to KDEL receptors of the mammalian target cell [15]. Therefore, it has been proposed that RTB binds to resident luminal ER proteins and is then transported piggyback into the ER [2]. After recognition by Edem1, ricin is presumably retrotranslocated into the cytosol, most likely by using the Sec61 translocon of the ER membrane [16,17,18]. After ER exit, a limited number of RTA molecules are somehow capable of escaping proteasomal degradation reachingtheir final target and causing cell death [16,19].

Despite our detailed knowledge on RTA *in vivo* toxicity, comparatively little is known about the intracellular toxin transport and the cellular components involved in this process. A deeper mechanistic understanding of toxin trafficking could not only help to design more effective antidotes and immunotoxins, it would also foster development of novel therapeutic strategies for the treatment of various human diseases including cancer [20,21,22]. 

The focus of our present study was to develop a yeast-based bioassay which would allow *in vivo* analyses of toxin uptake and transport in more detail. Previous studies already demonstrated that yeast ribosomes are highly sensitive to, and depurinated by, RTA [23]; however, so far all these studies have been performed by artificial RTA expression in the ER lumen. Therefore, analysis of intracellular toxin transport has largely been restricted to the analysis of toxin retrotranslocation from the ER into the cytosol [24]. Based on the observation that the addition of the mammalian-specific ER retention signal KDEL increases *in vivo* toxicity of ricin up to 250 fold [25,26], we asked if the addition of a yeast-specific ER retention signal (HDEL) to RTA likewise exhibits *in vivo* toxicity against HeLa cells. As we observed similar cytotoxicity for both toxin variants, RTA^HDEL^ and RTA^KDEl^, we built-up a yeast-based bioassay for the analysis of RTA uptake and intracellular transport. Further studies with this novel test system should shed more light on ricin trafficking *in vivo*, thereby helping to identify host cell proteins that are mechanistically involved in the intoxification by microbial and plant A/B toxins.

## 2. Materials and Methods

### 2.1. *Escherichia Coli* Strains, Plasmids, Culture Media and Genetic Techniques

Standard molecular manipulations were performed as described by [27]. *E. coli* TOP10 (*F’mcrA* Δ (*mrr*-*hsdRMS*-*mcrBC*) Φ80*lacZ*Δ*M15* Δ*lacX*74 *recA*1 *araD*139 Δ (*ara*-*leu*) 7697 *galU galKrpsL* (*StrR*) e*ndA*1* nupG*) was used for cloning. Using pRS316-RTA as template, RTA(His)_6_, RTA(His)_6_HDEL and RTA(His)_6_KDELwere PCR amplified in the presence of HiFi polymerase (Roche) and the following primer pairs: 5'-RTA (5'-GGATCCATGATAATTCCCAAACAATACCCAATTATAAACTTTAC) and 3'-RTA(His)_6_ (3'-AAGCTTGTCGACTTAATGATGATGATGATGATGAAACTGTGACGATGGTGGAGGTGC) for RTA(His)_6_, 5'-RTA and 3'-RTA(His)_6_HDEL (3'-AAGCTTGTCGACTTACAGTTCATCATGATGATGATGATGATGATGAAACTGTGACGATGGTGGAGGTGC) for RTA(His)_6_HDEL, and 5'-RTA and 3-RTA(His)_6_KDEL (3'-AAGCTTGTCGACTTACAGTTCATCTTTATGATGATGATGATGATGAAACTGTGACGATGGTGGAGGTGC) for RTA(His)_6_KDEL. Each primer introduced either a 5'*Bam*HI or 3'*Hind*III/*Sal*I cleavage site (underlined). After amplification, the corresponding DNA fragment was cloned into pSTBlue-1 (Novagen), sequenced and finally cloned as *Bam*HI/*Hind*III fragment into pET24a^(+)^ (Novagen) to obtain the expression vectors pET-RTA, pET-RTAHDEL and pET-RTAKDEL, respectively. All constructs were subsequently transformed into *E. coli* BL21 (DE3) (*F^−^ ompT gal dcm lon hsdS_B _*(*r_B_^−^m_B_^−^*) *λ *(*DE3* [*lacI lacUV5-T7 gene 1 ind1 sam7 nin5*], Stratagene) and after cells had reached an optimal density (OD_600_≈0.8–1), expression of each RTA variant was induced in the presence of 1 mM IPTG for 2 h at 28 °C. Cells were harvested, washed twice with sterile water and resuspended in binding buffer (500 mM NaCl, 10 mM imidazol and 20 mM KH_2_PO_4_) for subsequent RTA purification. Cell debris was removed and the supernatant was collected for Ni^2+^-NTA affinity chromatography. Sonicated supernatants were immediately used for SDS–PAGE, western analysis and/or Coomassie blue staining.

### 2.2. Yeast Strains and Culture Media

The *S. cerevisiae* wild-type strain BY4742 (MATα *his3*Δ1, *leu2*Δ0, *lys2*Δ0, *ura3*Δ0) and its isogenic knock-out mutants YNL084C (∆*end3*) and YDR418W (∆*rpl12B*) were obtained from Open Biosystems. Yeast cells were grown in YEPD (2% glucose, 2% peptone and 1% yeast extract) at 30 °C. For spheroplast preparation, each strain was grown in YEPD to late exponential phase (4–6 ×10^7^ cells per mL), harvested at 8000 rpm and washed twice with sterile water. Subsequently, 1 × 10^9^ cells were resuspended in 100 mL heroplast buffer (0.8 M sorbitol, 10 mM Tris-HCl (pH 7.5), 10 mM CaCl_2_, 2 mM DTT and 200 µg/mL zymolyase 20 T), incubated at 30 °C for 75 min, harvested at 4 °C and 2000 rpm and washed twice with incubation buffer (0.8 M sorbitol, 10 mM Tris-HCl (pH 4.7), 10 mM CaCl_2_,10 mM glucose).

### 2.3. Affinity Purification of RTA

Sonicated supernatants of *E. coli* clones expressing the (His)6-tagged RTA variants were applied onto a 5 mL HisTrap FF column (GE Healthcare) and eluted in a single step by the addition of imidazol (500 mM imidazol, 500 mM NaCl, 20 mM KH_2_PO_4_). Eluted protein fractions were desalted and equilibrated either in PBS (pH 7.4) for studies on mammalian cells or in incubation buffer for yeast experiments. Ni^2+^-NTA purified supernatants of *E. coli* expressing the empty vector pET24a^(+)^ without RTA served as negative control. After concentration through 10 kDa cut-off spin columns (Sartorius, Viva Spin 20), purified proteins were stored at 4 °C. Coomassie staining was employed to analyze protein purity and the level of protein expression was verified by western blot analysis. Total protein content was determined by using a BCA protein assay kit (Pierce).

### 2.4. Western Analysis and Protein Staining

After RTA expression, *E. coli* supernatants and Ni^2+^-NTA purified fractions were analyzed by SDS-PAGE by separating protein samples in 15% Tris-tricine SDS polyacrylamide gels [28]. After electrotransfer to PVDF membranes, blots were incubated with a polyclonal antibody against the ricin A subunit (diluted 1/1000). Thereafter, blots were treated with monoclonal peroxidase-coupled anti-sheep antibody (Sigma, diluted 1/13,000) and developed with Western lightning Plus ECL (PerkinElmer). Signals were detected with ChemiDoc XRS (BioRad). For Coomassie blue staining, SDS gels were incubated in a staining solution (0.1% (w/v) Coomassie blue R, 30% (v/v) methanol and 10% (v/v) acetic acid) for 2h and thereafter destained in a solution containing 30% (v/v) methanol and 10% (v/v) acetic acid [29].

### 2.5. Phase Contrast Microscopy

HeLa S3 cells were seeded in 24 well plates at a density of 1 × 10^5 ^ cells per well in DMEM medium containing 10% FCS and 1% penicillin-streptomycin and incubated at 37 °C in the presence of 5% CO_2_ for 18 h. After an additional incubation in the presence of purified RTA and/or control samples for 24 or 48 h, phase contrast microscopy was performed using an Olympus IX70 microscope under standard settings. For trypan blue staining, DMEM medium was removed, cells were stained in 0.4% trypan blue (Sigma) for 5 min, washed with PBS and analyzed under the microscope and counted in an automated cell counter (Invitrogen). For counting, cells were harvested 24 or 48 h after RTA treatment. Sample supernatants were collected, adherent growing cells trypsinated and pooled. Subsequently, pooled supernatants were harvested at 2000 rpm, washed once with PBS, resuspended in 100 µL PBS, and 100 µL 0.4% Trypan Blue were added. Cells were counted after 3 min of incubation.

### 2.6. Oyxgen-Sensor Microtiter Plate Bioassay

Wild-type yeast cells and selected knock-out mutants were grown in YEPD to exponential phase and spheroplasted by treatment with zymolyase 20 T as described above. Yeast spheroplasts (1.5 × 10^7^ cells) were seeded into 96 well oyxgen-sensor microtiter plates (Presence, round bottoms). Various concentrations of RTA variants were adjusted to a final volume of 200 µL incubation buffer per well. In each measurement, a two point calibration was conducted. The *k*_100_ value was measured with air saturated water and the *k*_0_ value with water containing 0.1% Na_2_SO_3_ solution. Oyxgen concentration was measured every 20 min over a time window of 16 h. Each sample measurement was performed in triplicate at 30 °C, 120 rpm and with a shaking diameter of 1 mm. Measurements were carried out in a fluorescence reader equipped with an integrated shaker (Fluoroskan Ascent, Labsystems, Vantaa, Finland) [30]. Oyxgen concentration and *p*O_2_ values were calculated according to the equations shown in (1) and (2).


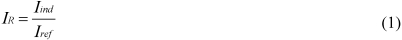



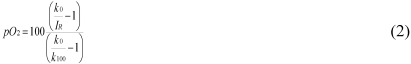


### 2.7. Cytotoxicity Assay

Yeast wild-type cells and knock-out mutants were cultivated and spheroplasted as described above. At a density of 1.5 × 10^7^ cells, intact cells and/or spheroplasts were incubated in black 96 well plates (Nunc) in the presence of each RTA variant in incubation buffer at 30 °C and low shaking (115 rpm) for 24 h. Measurements were performed using a PARADIGM (Beckham Coulther, Cartridge Multimode). Subsequently, cells were treated with 2.5 µL propidium iodide (PI, 1 mg/mL) for 3 min and fluorescence was measured at an extinction wavelength of 535 ± 25 nm and an emission wavelength of 635 ± 25 nm for 140 ms. Fluorescence was expressed as shown in (3).


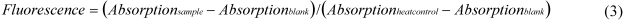


All experiments and measurements were repeated five times (*n* = 5) and the corresponding standard deviations are indicated for each experiment.

### 2.8. Cell Culture

HeLa S3 cells were obtained from ATCC and maintained in DMEM medium supplemented with 10% FCS and 1% penicillin-streptomycin. For cell vitality assays (TOX2, XTT based, Sigma), HeLa cells were seeded in 24 well plates at a density of 1 × 10^5^ cells per well and incubated in DMEM medium at 37 °C in the presence of 5% CO_2_ for 18 h. Thereafter, HeLa cells were incubated in the presence of increasing concentrations of purified RTA in DMEM medium for another 24 or 48 h. Subsequently, cells were washed twice with PBS, centrifuged between each step at 1600 rpm and finally resuspended in DMEM without phenol red and FCS. Thereafter, XTT was added in a ratio of 20% of DMEM medium and cells were incubated at 37 °C for another 3 h. Absorbance of the samples was measured with a spectrophotometer Ultrospec 2100 pro (Amershan Biosciences) at a wavelength of 450 nm. Cytotoxicity was expressed as shown in (4).


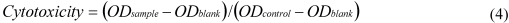


Each experiment was performed in triplicate (*n* = 3) and standard deviation is displayed in each figure. Statistical significance of the values was calculated by using the *t*-test method. 

## 3. Results and Discussion

### 3.1. RTA Expression and Purification

**Figure 1:**
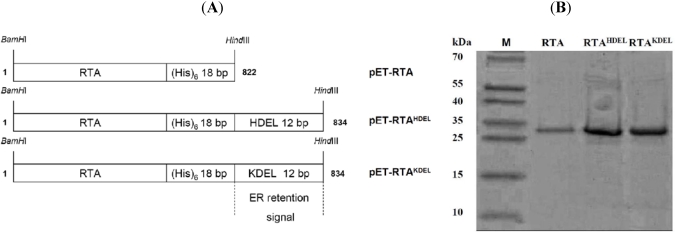
(**A**) Schematic outline of ricin toxin A (RTA) variants used in this study.In each fusion protein, the type of endoplasmic reticulum (ER) retention signal fused to RTA (K/HDEL) and the total size (in bp) is indicated. Length of the (His)_6_-tag and the mammalian- or yeast-specific ER retention motifs are also shown. RTA fusions were cloned into pET24a^(+)^ and expressed in *E. coli*; (**B**) RTA purification by Ni^2+^-NTA chromatography.Purified samples (15‑25 µg) were analyzed by SDS‑PAGE and Coomassie staining. Lane 1: PAGE Ruler prestained (Fermentas); Lane 2: unmodified RTA; Lane 3: RTA^HDEL^; Lane 4: RTA^KDEL^.

For recombinant ricin expression in *E. coli*, each RTA variant was PCR-amplified from plasmid pRS316K2_SP_-RTA [31] by adding *Bam*HI and *Hind*III cleavage sites to the 5'- and 3'-end, respectively. The resulting *Bam*HI/*Hind*III fragments were ligated into pET24a^(+)^ to obtain the expression vectors pET-RTA, pET-RTAKDEL and pET-RTAKDEL (Figure 1A). As His-tagged RTA variants had already been successfully expressed in *E. coli* [25,32], we used similar expression conditions for our constructs. In sonicated supernatants of the corresponding *E. coli* transformants strong protein bands at approximately 31 kDa were detectable by Coomassie staining which nicely matched the calculated size of RTA (31.0 kDa), RTA^HDEL^ (31.5 kDa) and RTA^KDEL^ (31.5 kDa) (Figure 1B). As expected, no RTA signal was detectable in the vector control and in cells cultivated under non-inducing conditions (data not shown). To concentrate and purify the protein after Ni^2+^-NTA affinity chromatography, each column fraction was analyzed by SDS-PAGE and Coomassie staining or western analysis probed with anti-RTA. Based on total protein content, all RTA variants were judged to be 90–95% pure with an overall yield of 40–50 mg/L RTA (Figure 1B).

### 3.2. RTA Containing a Mammalian or Yeast Specific ER Retention Signal is *in vivo* Toxic

**Figure 2:**
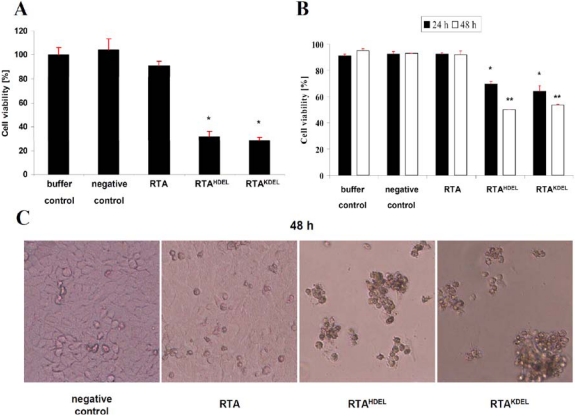
RTA containing a mammalian- or yeast-specific ER retention signal is toxic to HeLa cells. (**A**) XTT based cell viability assay of HeLa cells. Cells were incubated in the presence of 12 µg of the indicated RTA variant and controls for 48h (buffer control set to 100%). Each experiment was performed in triplicate (*n* = 3) and standard deviation (red bar) and *p*-values (*p< 0.05) are indicated; (**B**) Trypan blue staining of HeLa cells treated with 12 µg of each toxin variant after an incubation of 24 (black) or 48 (white) hours. Schemata show HeLa cell viability in % and the standard deviation for each sample (red bar). Each measurement was performed twice (*n* = 2). For *p*‑value calculation, RTA-treated samples were compared to the negative control (*p < 0.05; **p < 0.01); (**C**) Phase contrast microscopy of RTA treated HeLa cells after 48 h (10,000 magnification). Cells were incubated in the presence of 12 µg of the indicated RTA protein fusion.

To confirm that RTA variants expressed in *E. coli* are biologically active against mammalian cells, XTT cell viability assays were performed in which mitochondrial dehydrogenase activity of HeLa S3 cells was measured as an indirect means of cell viability. To ensure that the cells are not negatively affected by endogenous *E. coli* proteins, a negative control was included corresponding to NTA-purified proteins from *E. coli* clones expressing the empty vector. As shown in Figure 2A, no toxic effect was detectable in either untreated or negative control samples, whereas cell viability significantly declined 24 h after treatment with either RTA^KDEL^ or RTA^HDEL ^ (data not shown); after toxin treatment for 48 h, loss in cell viability further increased to 71.4% for RTA^KDEL^ and 68% for RTA^HDEL^ (Figure 2A), thereby confirming previous reports on RTA^KDEL^ toxicity against mammalian cell lines such as HeLa, MCF, Jurkart and Vero [26,32]. Unexpectedly however, we did not observe any significant decrease in cell viability after treatment with unmodified RTA at or below concentrations of 12 µg RTA (Figure 2A; data at lower toxin concentrations are not shown). The fact that unmodified RTA is non-toxic on mammalian cells confirms recent data of Wang and colleagues [33]. In contrast, *in vitro* protein inhibition studies of Wales *et al.* demonstrated that unmodified RTA is active at high toxin concentration, however its inhibitory potential against HeLa cells was up to 10-fold lower compared to RTA^KDEL^[25]. Our present study confirms these data as we also observe significant *in vivo* toxicity of non-modified RTA at toxin concentrations >80 µg after 48 h (data not shown). We thus assume that cells treated with low doses of RTA are being inhibited in protein biosynthesis; however this inhibition is not sufficient to induce *in vivo* cell death. This might explain the observed lack in RTA toxicity under low toxin concentrations in XTT viability assays. To our knowledge, we showed here for the first time that the addition of a yeast specific ER retention signal (HDEL) to RTA results in a comparably strong cytotoxic effect to what can be observed for RTA^KDEL^ on HeLa cells. Dose response curves and *p*-value calculations of both constructs did not show any significant difference in the presence of 0.1 to 80 µg toxin (data not shown). One possible explanation for the increased cytotoxicity of RTA^HDEL^ could be that mammalian cells—in contrast to yeast—possess three different ER retention signal recognizing KDEL receptors. One of them (Erd23) has been shown to recognize HDEL carrying proteins and to catalyze subsequent retrograde transport back to the ER [34]; in this study it was demonstrated that Erd21 preferentially recognizes KDEL, whereas Erd23 mainly binds proteins with a *C*-terminal HDEL motif; our present cell viability data are consistent with these findings. To fortify the cell viability data, we looked at the morphology of HeLa cells before and after RTA treatment (Figure 2C). Negative control cells did not show any visible change in morphology compared to untreated or buffer treated cells (data not shown). The majority of cells grew adherent on the bottom of the well and showed normal growth behavior; only a minimal amount of detached cells was detectable. Samples treated with unmodified RTA showed the same unchanged phenotype as negative control cells (Figure 2C); this result was also confirmed by the numbers of trypan blue positive cells which likewise was not altered in RTA‑treated cells (Figure 2B). In contrast, HeLa cells treated with either RTA^HDEL^ or RTA^KDEL^ showed significant higher numbers of detached cells at 24 h, while after 48 h most of the cells grew non-adherent and were detached from the bottom of the well (Figure 2C). Moreover, trypan blue staining of cells treated with RTA^HDEL^ or RTA^KDEL^ identified a high number of dead cells compared to negative control cells or cells treated with unmodified RTA (Figure 2B). In these samples, cell numbers were reduced over 50% compared to RTA lacking an ER retention motif (data not shown). So, in total, our data from cell viability assays, trypan blue staining and phase contrast microscopy altogether demonstrate that the addition of both, HDEL or KDEL to RTA results in a significant increase in the *in vivo* toxicity against HeLa cells. 

### 3.3. Recombinant RTA Variants Induce Cell Death in Yeast Spheroplasts

**Figure 3:**
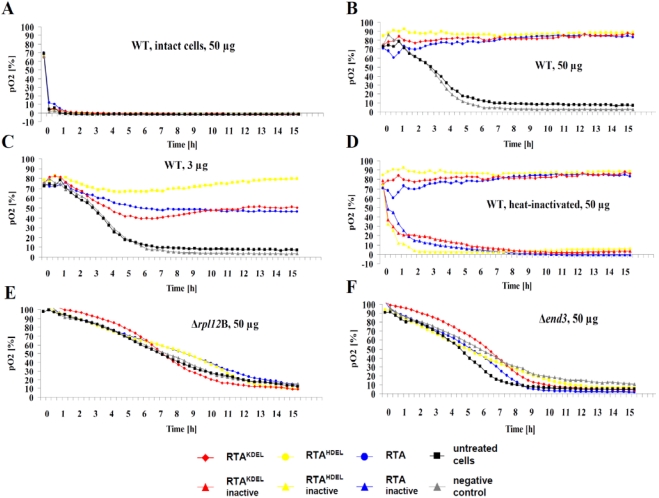
RTA toxicity against yeast spheroplasts. Dissolved oxygen concentration was measured for intact wild-type cells (WT) and spheroplasts in the presence of the indicated RTA variant. All experiments were performed in triplicate (*n* = 3) at 30 °C and 120 rpm over 16 h. (**A**) Intact wild-type yeast cells treated with 50 µg of the indicated RTA variant; (**B**,**C**) Same experiment performed on yeast spheroplasts in the presence of 50 or 3 µg RTA; (**D**) Spheroplasts treated with 50 µg RTA before and after heat-inactivation; (**E**,**F**) Yeast spheroplasts of a Δ*rpl12B* or Δ*end3* mutant in the presence of 50 µg of the indicated RTA variant.

In further experiments we wanted to analyze whether or not RTA, RTA^HDEL^ and/or RTA^KDEL^ also influence cell viability in yeast. Since yeast cells do not possess terminal galactose residues within their cell wall components, native ricin cannot enter yeast cells because potential RTB binding sites are lacking on the yeast cell surface [15]. Therefore, all studies on intracellular RTA transport in yeast had so far been restricted to the analysis of toxin retrotranslocation from the ER into the cytosol by expressing RTA variants in the ER [16,24,31]. Because of this restriction in using yeast as model to study toxin uptake and trafficking, we aimed to establish a modified yeast system by-passing these limitations. Such a system would have a big advantage over mammalian cells as an entire collection of yeast knock-out mutants is available allowing a comprehensive genetic screening to identify genes whose products are directly or indirectly involved in intracellular RTA transport. Potential candidates identified in yeast can then be analyzed for their particular function in mammalian cells. With such an assay, we could look at the ER upstream transport of RTA from the cell membrane to the ER. To bypass the lack of ricin binding sites in the cell wall of yeast, we generated yeast spheroplasts by zymolyase treatment and subsequently performed oxygen sensor microtiter plate assays to analyze yeast sensitivity against RTA [30]. If RTA was toxic for yeast spheroplasts, dissolved oyxgen concentration would be expected to stay constant. As illustrated in Figure 3A, we indeed observed such an effect in spheroplasts, while intact yeast cells showed the expected rapid decrease in dissolved oxygen, corresponding to the effect seen in the negative controls. The experiments with yeast spheroplasts uncovered an additional important finding: RTA, RTA^HDEL^ and RTA^KDEL^ likewise showed similar constant and high levels in dissolved oxygen at concentrations of about 12–50 µg RTA (Figure 3B), while the negative control sample showed a similar kinetic in oyxgen consumption as untreated spheroplasts. Compared to intact cells, yeast spheroplasts showed slower oxygen uptake over time, resulting in a shift seen in the time course (Figure 3B) which is likely caused by a slower growth of yeast spheroplasts which hardly double during the time course of the experiment. Nevertheless our data support the hypothesis that RTA is taken up by yeast spheroplasts and finally reaches the cytosol to inhibit protein biosynthesis. In contrast, the cell wall of intact yeast prevents toxin uptake and therefore causes RTA resistance. The current data also shows that RTA lacking a *C*-terminal retention signal is biologically active against yeast spheroplasts, while it completely loses its *in vivo* toxicity after heat-treatment at 95 °C for 20 min (Figure 3D). As no significant difference in dissolved oxygen concentration was seen against the three tested RTA variants at high toxin concentration, we analyzed RTA toxicity on yeast spheroplasts at low toxin concentration. As illustrated in Figure 3B, RTA^HDEL^ resulted in a mean average of 79.8% dissolved oxygen after 16 h which was significantly higher than dissolved oxygen concentrations present in samples that had been treated with either RTA (47.1%) or RTA^KDEL^ (50.5%); both variants—RTA and RTA^KDEL^—showed similar kinetics at a low toxin concentration of 3 µg (Figure 3C). *p*-values below 0.05 and 0.01 confirmed significance of the observed effect in comparison to RTA^HDEL^ (data not shown). Taken together, these results indicate that the yeast-specific ER retention motif HDEL is more efficient in ensuring retrograde RTA transport than the mammalian-specific retention signal KDEL. This might be explained by the fact that yeast—in contrast to mammals—only possess a single cellular HDEL receptor (Erd2p) which preferentially recognizes and binds HDEL-carrying proteins within the secretory pathway [35,36].

### 3.4. RTA Endocytosis is an Essential Prerequisite for Cell Killing

Since endocytotic uptake of only a limited number of toxin molecules is sufficient for *in vivo* cell killing [37], we asked if spheroplasts of a yeast Δ*end*3 mutant which is blocked in early steps of both, fluid-phase and receptor endocytosis [38] behave phenotypically RTA resistant when tested under the same conditions as wild-type cells. As illustrated in Figure 3F, this was indeed the case and Δ*end*3 cells were effectively protected against all three toxin variants RTA, RTA^KDEL^ and RTA^HDEL^. The same resistant phenotype was also seen in spheroplasts of a Δ*rpl12B* mutant (Figure 3E) which expresses a mutant ribosomal protein of the 60S subunit that prevents ricin-mediated 28S rRNA depurination within the sarcin/ricin loop [39].

**Figure 4:**
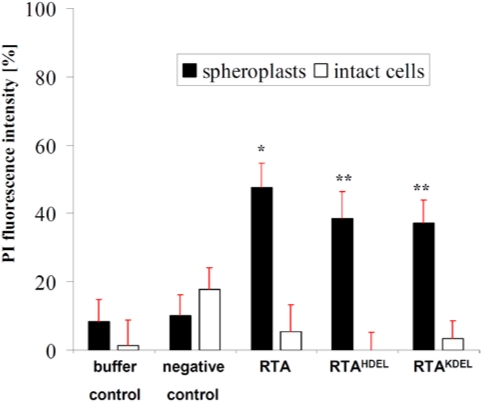
PI fluorescence of RTA-treated intact cells and yeast spheroplasts. Propidium iodide (PI) fluorescence intensity of yeast spheroplasts and intact cells after treatment with the indicated RTA variant. In the positive control (set to 100%), cells were heat-inactivated at 95 °C for 20 min. In each case, cells were incubated in the presence of 12 µg of the indicated RTA variant for 24 h (mean average of five independent experiments; standard deviation (red bar) and *p*-values (*p< 0.05, **p< 0.01) are indicated).

To further confirm the power and significance of the yeast-based RTA toxicity assay described here, we additionally performed propidium-iodide (PI) staining of yeast spheroplasts and determined PI fluorescence intensity after RTA treatment. As summarized in Figure 4, a significant increase in fluorescence was only seen in yeast spheroplasts (and not in whole cells) after RTA treatment at high toxin concentration (12 µg), thereby nicely confirming our previous results from oxygen consumption measurements (see Figure 3B). 

## 4. Conclusions

In the present study we demonstrate that both the addition of a mammalian- and/or a yeast-specific ER retention signal (HDEL, KDEL) to the *C*-terminus of RTA significantly increases its *in vivo* toxicity against mammalian cells and yeast cell spheroplasts. Furthermore, we established an oxygen sensor based bioassay which now opens the possibility to use yeast as model to study A/B toxin uptake and intracellular trafficking in more detail. We believe that this bioassay will be helpful to dissect and mechanistically understand how microbial, plant and viral A/B toxins can efficiently intoxicate mammalian cells. Such comprehensive screening in yeast might also bring up novel targets and therapeutic strategies for the treatment of various human diseases, including cancer.

## Acknowlegments

We are grateful to Lynne Roberts for kindly providing the ricin A antibody and the plasmid pRS316-K2_SP_-RTA. Special thanks to Klaus Witte for technical assistance in the affinity purification of recombinant RTA variants, and to the members of the Schmitt lab for helpful discussions, especially to Nina Müller, Thorsten Hoffmann, Esther Gießelmann, Björn Diehl and Frank Breinig. BB was kindly supported by a PhD fellowship from Saarland University. This study was in part supported by a grant from the Deutsche Forschungsgemeinschaft to MJS.
